# Molecular Mechanisms of *Leonurus Cardiaca* L. Extract Activity in Prevention of Staphylococcal Endocarditis—Study on in Vitro and ex Vivo Models

**DOI:** 10.3390/molecules24183318

**Published:** 2019-09-12

**Authors:** Beata Sadowska, Dariusz Laskowski, Przemysław Bernat, Bartłomiej Micota, Marzena Więckowska-Szakiel, Anna Podsędek, Barbara Różalska

**Affiliations:** 1 Department of Immunology and Infectious Biology, Institute of Microbiology, Biotechnology and Immunology, Faculty of Biology and Environmental Protection, University of Lodz, Banacha 12/16, 90-237 Lodz, Poland; bartlomiejmicota@o2.pl (B.M.); marzena.wieckowska@biol.uni.lodz.pl (M.W.-S.); barbara.rozalska@biol.uni.lodz.pl (B.R.); 2 Department of Microbiology, Faculty of Biology and Environmental Protection, Nicolaus Copernicus University in Torun, Lwowska 1, 87-100 Torun, Poland; laskosd@umk.pl; 3 Department of Industrial Microbiology and Biotechnology, Institute of Microbiology, Biotechnology and Immunology, Faculty of Biology and Environmental Protection, University of Lodz, Banacha 12/16, 90-237 Lodz, Poland; przemyslaw.bernat@biol.uni.lodz.pl; 4 Institute of Technical Biochemistry, Faculty of Biotechnology and Food Sciences, Lodz University of Technology, Stefanowskiego 4/10, 90-924 Lodz, Poland; anna.podsedek@p.lodz.pl

**Keywords:** *Leonurus cardiaca* L., infective endocarditis, blood platelets, *Staphylococcus aureus*, microbial adhesion, cell–pathogen interaction

## Abstract

Better understanding the mechanisms of *Leonurus cardiaca* L. extract (LCE) activity is necessary to prepare recommendations for the use of LCE-based herbal products for preventive/supportive purposes in case of infective endocarditis (IE) and other staphylococcal invasive infections. The aim of the study was to analyze molecular mechanisms of LCE effect on *Staphylococcus aureus* and blood platelets in the context of their interactions playing a pivotal role in such disorders. Using atomic force microscopy, we demonstrated that adhesion forces of *S. aureus* were markedly reduced after exposure to LCE at subinhibitory concentrations. The effect resulted from the impact of LCE on *S. aureus* cell morphology and the composition of phospholipids and fatty acids in bacterial membranes (assessed by HPLC), which modulated their stabilization, hydrophobicity, and charge. Moreover, using FACS we showed also that LCE significantly reduced GP IIb/IIIa expression on blood platelets, thus the disruption of platelet-fibrinogen interactions seems to explain antiplatelet effect of LCE. The obtained results prove the usefulness of LCE in the prevention of *S. aureus* adhesion, platelet activation, and vegetations development, however, also pointed out the necessity of excluding the cationic antibiotics from the treatment of *S. aureus*-associated IE and other invasive diseases, when motherwort herb is used simultaneously as an addition to the daily diet.

## 1. Introduction

Infective endocarditis (IE) is an acute, life-threatening disease, in which microbial colonization leads to progressive damage of heart tissue (usually valves) via the formation of complex aggregates called vegetations containing microbes, fibrin, activated platelets, and phagocytes. The most common etiological agents of IE include *Staphylococcus aureus*, oral streptococci from *Streptococcus* Viridans group, *Enterococcus* spp., and coagulase-negative staphylococci (CNS). Although many bacteria and even fungi, if present in the bloodstream, may colonize heart tissue or the biomaterials placed in it (e.g., artificial valves, stents, pacemakers), *S. aureus* seems to be best adapted to settle both normal and damage endocardium thus it is responsible for 27–32% of IE cases [1,2,3]. The deposition of the microorganisms into the vegetations (usually as biofilm form) depends on their affinity for adhesion. Many surface molecules called MSCRAMMs (microbial surface components recognizing adhesive matrix molecules), including clumping factor A (ClfA), fibronectin binding proteins (FnBPs), staphylococcal protein A (SpA), and secretory proteins such as coagulase (Coa) or von Willebrand factor binding protein (vWbp) participate in *S. aureus* adherence, biofilm formation, and myocardial/endovascular diseases development [2,4,5]. Due to their complex structure and physiology, biofilms enhance microbial cell survival under environmental stress conditions, including exposure to antimicrobials and the components of host immune system. Therefore, biofilm-associated infections (BAI) are hard to treat and frequently require the removal of tissues or biomaterials colonized by the microorganisms, which prolongs hospitalization and elevates the costs of treatment [6,7,8]. There is no doubt that it is much better to prevent biofilm formation than to cure a BAI. However, because of antibiotic therapy shortage (the 21st century is even called the “post-antibiotic era”), very high drug-resistance among microorganisms and the ability to induce resistance mechanisms, the usage of antibiotics for preventive purposes should be limited. Thus, looking for alternative methods to inhibit microbial adhesion and biofilm formation has recently attracted much attention. Some plant-origin products containing biologically active secondary metabolites such as phenolic compounds possess the properties interesting in this respect. According to Quave et al. [8], for instance, the fraction of butanol extract from the root of *Rubus ulmifolius* Schott rich in ellagic acid and its derivatives inhibited *S. aureus* biofilm formation. Methanolic extract from *Opuntia ficus-indica* (L.) Mill. reduced biofilm formation by *Escherichia coli* and *S. aureus* in a dose-dependent manner [9]. Kaiser et al. [10] demonstrated that the isothiocyanates mixture consisted of 38% (*v/v*) allylisothiocyanate, 50% benzylisothiocyanate, and 12% phenylethyl-isothiocyanate according to the proportion of nasturtium (*Tropaeolum majus* L.) and horseradish (*Armoracia rusticana* P. Gaertn., B. Mey. & Scherb.) in the phytomedical preparation Angocin, used at sub-MIC significantly reduced *Pseudomonas aeruginosa* biofilm biomass, bacterial metabolic activity, and proliferation. The extract from the leaves of *Eugenia uniflora* L. was able to reduce the adhesion of *Candida albicans* and non-*C. albicans* clinical isolates to human buccal epithelial cells, biofilm formation, and cell surface hydrophobicity [11]. Alternatively, originally obtained polyphenol-rich extract from *Leonurus cardiaca* L. (LCE) is the subject of our interest, since motherwort herb possesses known beneficial effects on the heart and circulatory system and is used to strengthen the heart muscle, regulate blood pressure and heart rhythm in such herbal products as Cardiosan, Cardionervit, or Cardiogran. In our previous work we demonstrated that *S. aureus* aggregate formation in plasma, microbial adherence to the deposit of fibrin network, plasma clotting by staphylocoagulase, as well as expression of virulence factors participating in *S. aureus* adhesion and biofilm formation (SpA, α-toxin) were negatively affected by LCE in a dose-dependent manner. On the other hand, staphylococci exposed to LCE also showed higher tolerance to exogenous hydrogen superoxide, which may help to avoid host immune response [12].

To confirm the legitimacy of LCE use in prevention or supporting the treatment of IE and other systemic infections caused by staphylococci, the molecular mechanisms of the effect of LCE on staphylococci–blood platelet interactions occurring in the growing vegetations were investigated in the present study. The direct impact of LCE on *S. aureus* adhesive properties using atomic force microscopy, lipid composition of staphylococcal cell membranes by HPLC, blood platelet adhesion to fibrinogen using colorimetric assay, and the expression of their important receptors with FACS were assessed.

## 2. Results and Discussion

**Figure 1:**
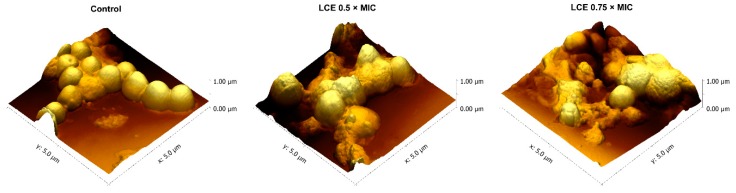
Atomic force microscopy (AFM) images of *S. aureus* 8325-4 control cells and the cells exposed on *L. cardiaca* L. extract (LCE) at 0.5 × MIC or 0.75 × MIC for 24 h.

**Figure 2:**
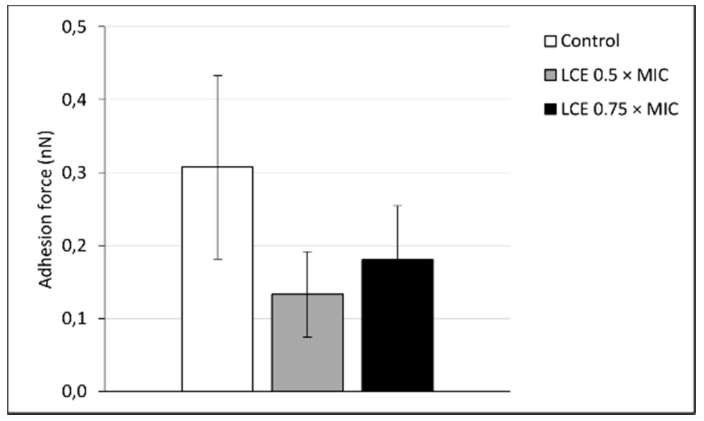
Changes of *S. aureus* 8325-4 adhesive properties after exposure to *L. cardiaca* L. extract (LCE) at 0.5 × MIC or 0.75 × MIC for 24 h, measured using atomic force microscopy (AFM). Statistical analysis was estimated with nonparametric Kruskal–Wallis one-way ANOVA test. The differences were significant in comparison to the control (*p* < 0.001).

**Figure 3:**
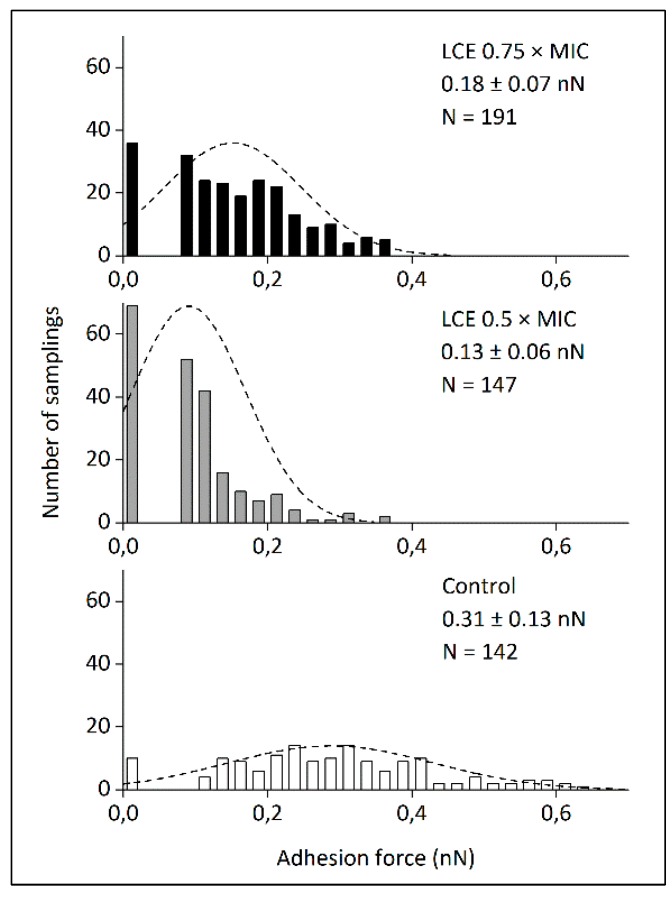
Adhesion force histogram obtained for *S. aureus* 8325-4 control cells and the cells exposed on *L. cardiaca* L. extract (LCE) at 0.5 × MIC or 0.75 × MIC for 24 h. Lines denote fitted function describing normal distribution of experimental points. Mean ± standard deviation. N—number of force–distance curve.

Phytochemical analysis of originally prepared *Leonurus cardiaca* L. extract (LCE) used in the present study was presented previously by Sadowska et al. [13] and demonstrated a high content of polyphenols (137.0 ± 0.8 mg/g) with hydroxycinnamic acid derivatives (81.3 ± 5.7 mg/g) as predominant phenolic compounds. Several researches, inclusive of our previous studies [12,13,14], showed that such secondary metabolites of plants exhibit a wide range of biological activity and may influence both physiological processes in human body (e.g., hemostasis, immune response, maintenance of physiological barriers of skin, mucosa, and endothelium) and host–pathogen interactions important for the course of infections. Motherwort herb is known to possess antihypertensive, heart-strengthening, antioxidant, analgesic, anti-inflammatory, neuroprotective, and antimicrobial effects [15,16]. In our previous studies we demonstrated unknown antiadhesive and anti-biofilm activity of LCE on staphylococcal infective endocarditis in vitro model. LCE used at sub-inhibitory concentrations (0.75 × MIC and 0.5 × MIC) negatively affected *S. aureus* adhesion to both native and conditioned with extracellular matrix proteins (ECM) surfaces, as well as to the deposit of fibrin network [12,14]. In the present study we confirmed these results testing adhesive properties of *S. aureus* by atomic force microscopy (AFM) (Figure 1). The force spectroscopy analysis revealed significant differences in the adhesive properties of *S. aureus* after exposure to LCE (*p* < 0.001) (Figure 2). Adhesion force were markedly reduced following LCE treatment at both concentrations, with a substantial increase (*ca.* 16–32%) in the number of curves exhibiting no adhesion (Figure 3). Interestingly, the highest decrease in adhesion force by 58% was observed for the cells exposed on LCE at lower concentration (0.5 × MIC). Thus, the adhesion properties of *S. aureus* were substantially affected by the LCE. Moreover, the effect of LCE treatment on *S. aureus* cell morphology was demonstrated (Figure 1). AFM is seen as a proper tool for the observation the impact of antimicrobial preparations on single microbial cell morphology to better understand the mechanisms of their activity [17,18]. In our study bacteria in native conditions existed mostly in clusters and showed the typical near-spherical shape of the cells. The untreated cells had a relatively smooth surface without pores or any ruptures in comparison with treated cells. Exposure to LCE caused the deformation of the cell wall, with a visible increase of in roughness. Moreover, some cells collapsed, indicating a loss of cellular content. The effect was tightly dependent on the LCE concentration, being stronger for higher concentration. However, observed morphological changes of staphylococcal cell surfaces can only partly explain antiadhesive properties of *L. cardiaca* L. extract, since LCE used at 0.5 × MIC was more potent in the reduction of *S. aureus* adhesion simultaneously exhibiting moderate effect on cell morphology compare to 0.75 × MIC.

**Figure 4:**
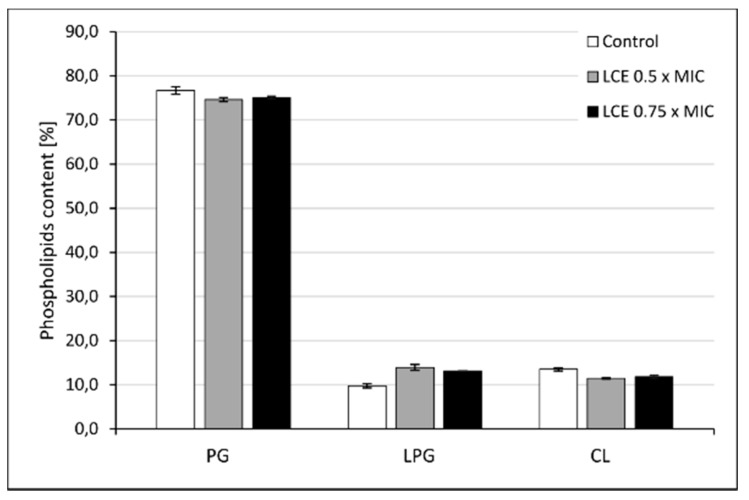
Composition of cell membrane phospholipid classes of control *S. aureus* 8325-4 cells or the cells treated with *L. cardiaca* L. extract (LCE) at 0.5 × MIC or 0.75 × MIC for 24 h, measured by HPLC–MS. PG—phosphatidylglycerol, LPG—lysyl-phosphatidylglycerol, CL—cardiolipin.

**Figure 5:**
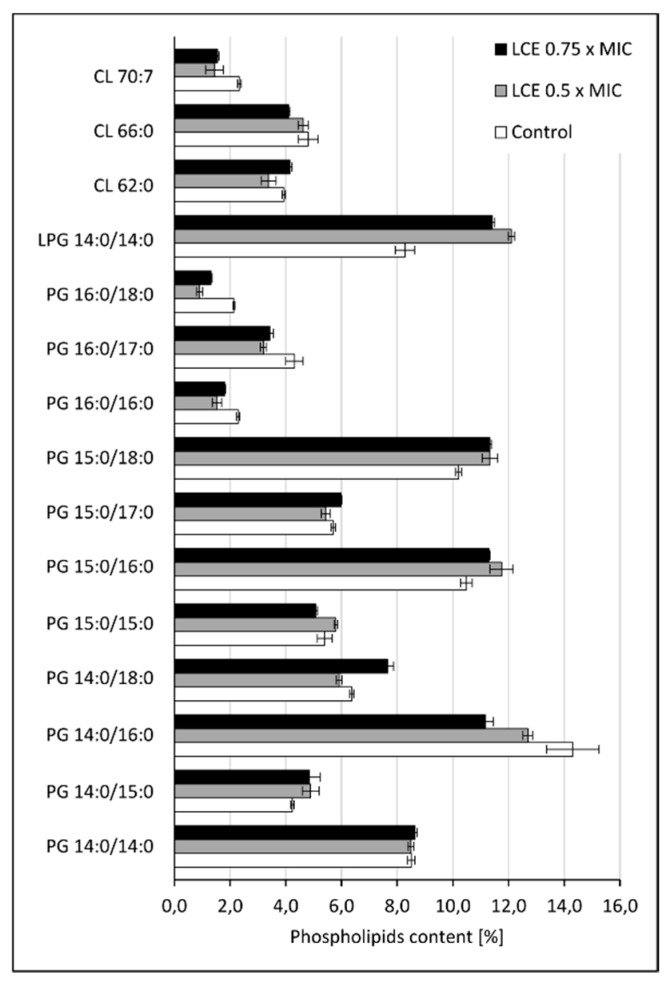
Content of the main phospholipid species (achieving above 2% in control) in cell membrane of control *S. aureus* 8325-4 cells or the cells treated with *L. cardiaca* L. extract (LCE) at 0.5 × MIC or 0.75 × MIC for 24 h, measured by HPLC–MS. PG—phosphatidylglycerol, LPG—lysyl-phosphatidylglycerol, CL—cardiolipin.

**Figure 6:**
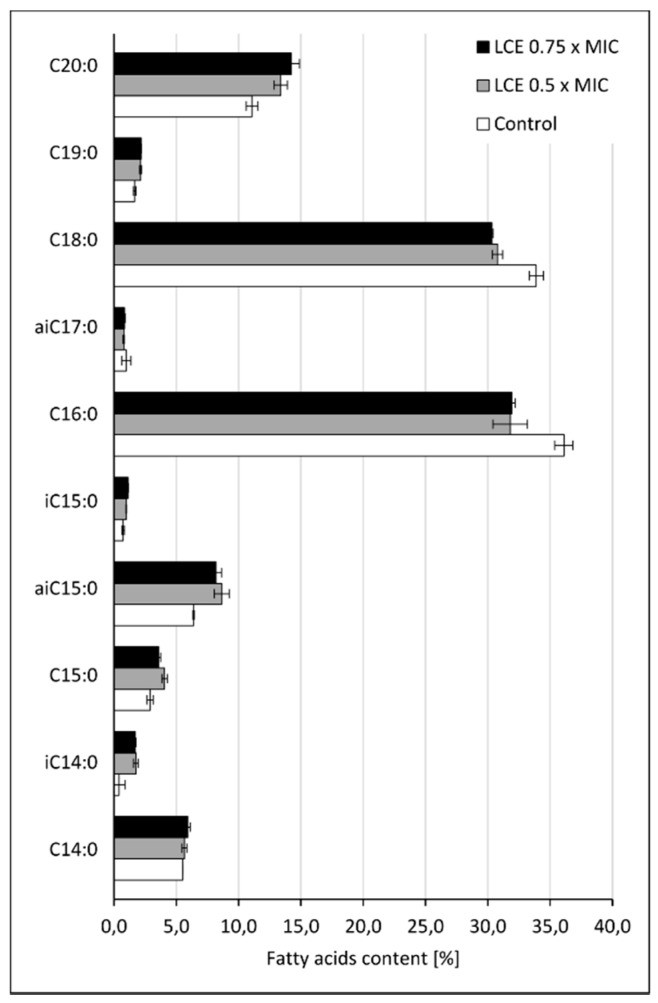
Composition of cell membrane fatty acids of control *S. aureus* 8325-4 cells or the cells treated with *L. cardiaca* L. extract (LCE) at 0.5 × MIC or 0.75 × MIC for 24 h, measured by HPLC–MS. iC/aiC—branched fatty acids with isomerism, respectively, iso/anteiso.

Cell-to-cell interactions, also including those between pathogens and host cells, as well as microbial adhesion to abiotic surfaces depend on many factors, such as physical forces, surface hydrophobicity, charge, diversity of microbial cell envelopes, expression of specific adhesins, and others [4,19,20,21]. Modifications in microbial cell wall and membrane composition may play an important role in those processes, too. To verify this hypothesis the effect of LCE on the profile of the lipids in *S. aureus* cell membrane using qualitative and quantitative tests were assessed. The cytoplasmic membranes of Gram-positive bacteria, including *Staphylococcus* spp., very commonly contain phosphatidylglycerols esterified with lysine, alanine, or ornithine [22,23]. The content of both phosphatidylglycerol (PG) and lysyl-phosphatidylglycerol (LPG), and also cardiolipin (CL), was assessed in our study. In *S. aureus* cells exposed to LCE the general level of LPG was growing at the expense of PG and CL as compared to control (untreated) cells (Figure 4), however these differences seem very small. Therefore, it is hard to say if observed modifications are sufficient to affect membrane function. On the other hand, it was reported that in staphylococci exposed to antimicrobial cationic peptides, the charge of membrane was modulated and an anionic PG was converted to cationic LPG. Such reaction was mediated by a membrane protein called the multiple peptide resistance factor F (MprF), which transfers an aminoacyl group from lysine-tRNA to PG [22,23]. The described modification is therefore the answer of staphylococci to stress conditions, so exposure to LCE seems to be treated by *S. aureus* as a stress factor. Moreover, analyzing the composition of the main phospholipid species (with content above 2%) in the cell membrane of control and LCE-treated *S. aureus* (Figure 5), an almost 0.5-fold increase in the level of LPG 14:0/14:0 was demonstrated in LCE-exposed samples. Although, for other phospholipids different directions of change were noticed. Small increase in the level of branched fatty acids (BCFAs) with isomerism anteiso (aiC15:0) as compared with control cells was also observed (Figure 6). A mixture of BCFAs and straight-chain fatty acids (SCFAs) additionally complicated by the presence of staphyloxanthin, a triterpenoid carotenoid with a C30 chain being one of important *S. aureus* virulence factors, are the main components of these bacteria membrane. BCFAs are produced *de novo* from the branched-chain amino acids, such as isoleucine (anteiso odd-numbered fatty acids), leucine (iso odd-numbered fatty acids), valine (iso even-numbered fatty acids), and the position of branching cannot be later modified. Thanks to this, BCFAs disrupt the close packing of fatty acyl chains, thus their presence increases the fluidity of cell membrane and prevents forming of crystal structures [24]. Therefore, it can be assumed that the observed changes in the composition of phospholipids and fatty acids in the presence of LCE, followed by membrane charge, fluidity, and hydrophobicity, at least partially determine the adhesive properties of *S. aureus* cells, leading to the desired effects such as reduction of adhesion forces measured using single-bacterial contact probe atomic force microscopy, and inhibition both of staphylococcal adhesion and biofilm formation, which we demonstrated previously [12,14]. On the other hand, the same modifications can cause the increased resistance of staphylococci to antimicrobial cationic peptides being important component of innate immunity or cationic antibiotics (e.g., daptomycin), by changing the charge of the bacterial surface [22,23]. Moreover, environmental factors, mainly chemical stress stimuli targeting the cell wall or membrane, but also mechanical stress as this triggered by cell adhesion and thus deformation of bacterial cell wall/membrane, can change staphylococcal genes expression by one- or two-component regulatory systems, finally limiting the effects of stress conditions and promoting the survival of microbial cells. It was shown, for instance, that the presence of nisin upregulates the NsaAB efflux pump by NsaRS two-component regulatory system for more effective efflux of the antimicrobial peptide from *S. aureus* cytoplasm [25]. We can assume that LCE may have a similar effect on genes expression of staphylococci, modifying diverse cell properties.

**Figure 7:**
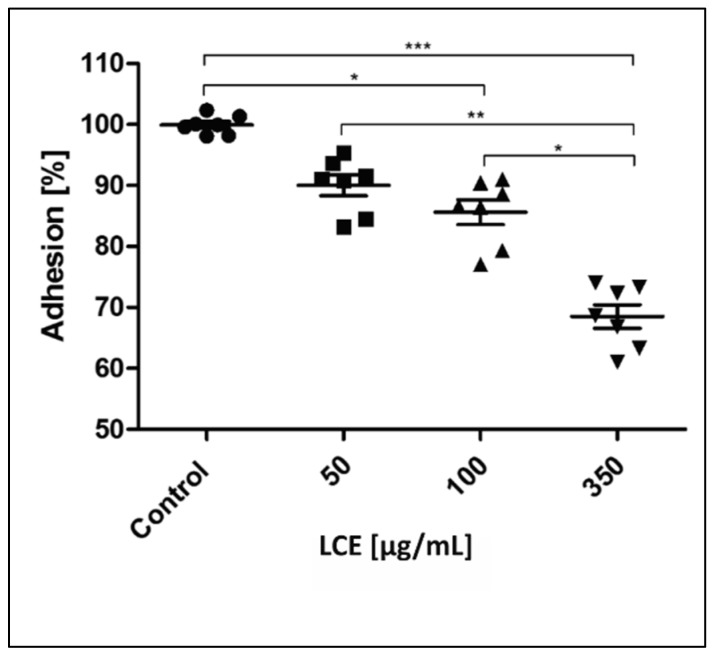
Effect of *L. cardiaca* L. extract (LCE) on human blood platelet adhesion to fibrinogen. The results are presented as a percentage of control for single individuals and expressed also as a median % ± SD. Statistical analysis was estimated with nonparametric Kruskal–Wallis one-way ANOVA test with Bonfferoni’s correction (significant differences: * *p* < 0.05; ** *p* < 0.01, *** *p* < 0.001).

**Figure 8:**
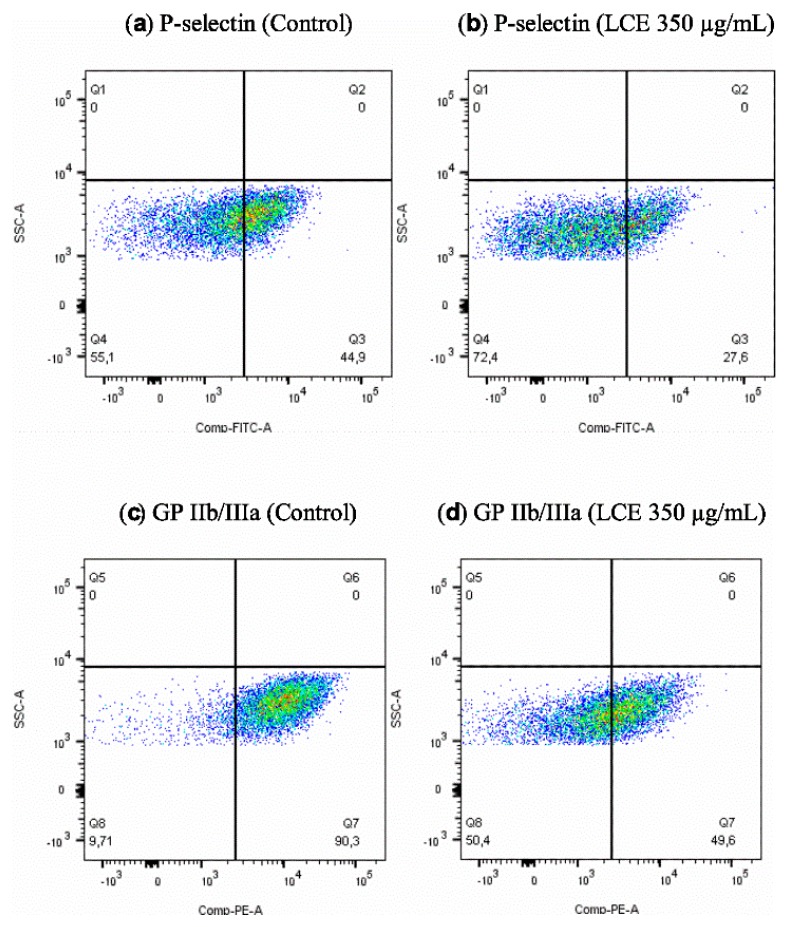
Representative dot plots for flow cytometry analysis. (**a**,**c**) Control: P-selectin and GP IIb/IIIa expression on platelet surface after ADP activation. (**b**,**d**) P-selectin and GP IIb/IIIa expression on platelet surface after ADP activation preceded by platelets co-incubation with *L. cardiaca* L. extract (LCE) used at 350 µg/mL.

**Figure 9:**
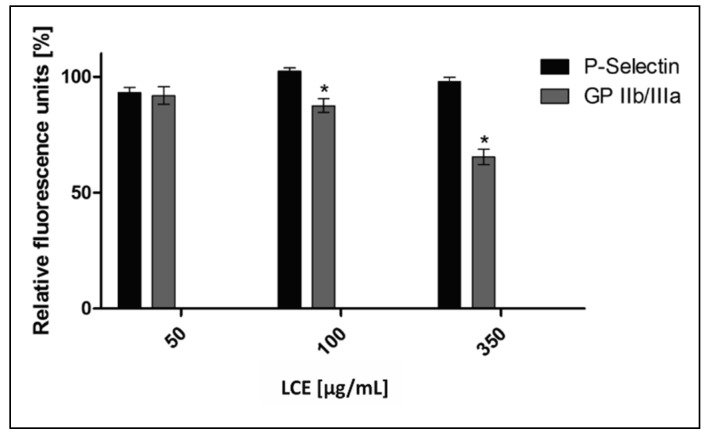
Effect of *L. cardiaca* L. extract (LCE) on human blood platelet receptor expression. The results are presented as a percentage of relative fluorescence unit (RFU) in comparison to control (RFU considered as 100%) and expressed as a mean % ± SEM. Statistical analysis was estimated with nonparametric Kruskal–Wallis one-way ANOVA test with Bonfferoni’s correction (significant differences: * *p* < 0.05).

**Figure 10:**
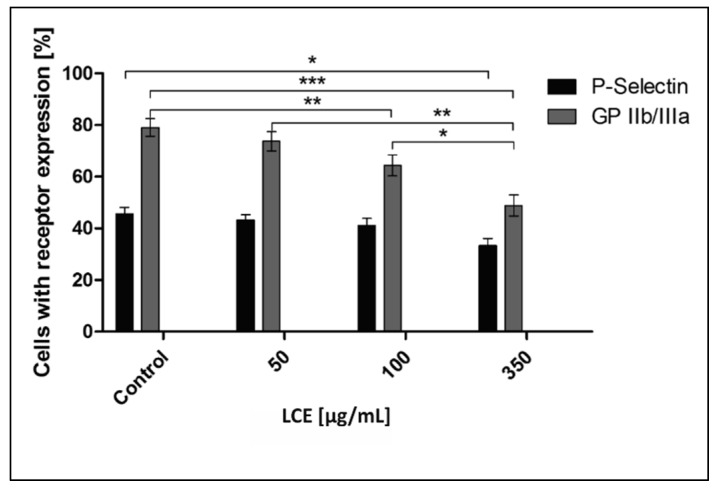
Effect of *L. cardiaca* L. extract (LCE) on human blood platelet activation. The results are presented as a percentage of activated cells (with P-selectin or GP IIb/IIIa exposure) in comparison to total amount of analyzed cells and expressed as the mean ± SEM. Statistical analysis was estimated with nonparametric Kruskal–Wallis one-way ANOVA test with Bonfferoni’s correction (significant differences: * *p* < 0.05; ** *p* < 0.01, *** *p* < 0.001).

The situation in vivo is much more complicated because of cell–cell, cell–protein, and cell–pathogen interactions. In the development of IE and some other cardiovascular diseases (CVD), such components as endothelium, platelets, immunocompetent cells, fibrinogen (Fg)/fibrin (Fb), physiological platelet agonists (e.g., adenosine diphosphate (ADP), arachidonic acid (AA), thrombin, collagen), and microorganisms are involved [1,3,26]. To define comprehensively an activity of *L. cardiaca* L. extract after potential oral administration the effect of LCE on blood platelets—the cells pivotal in IE pathogenesis—was assessed ex vivo. Both platelet adhesion to Fg and selected receptors expression (P-selectin, GP IIb/IIIa) on activated platelets were evaluated. Taking into consideration the limited intestinal absorption and availability in other tissues of plant-origin preparations after oral administration, as well as their bioconversion by intestinal microbiota [27,28], such high concentrations as previously used for bacteria, 4.5 mg/mL, which correspond with 0.75 × MIC and 3 mg/mL (0.5 × MIC), do not seem possible to achieve in tissue. Therefore, we decided to use much lower concentrations (50–350 µg/mL) to test the LCE impact on eukaryotic cells. It was shown that the adhesion of freshly isolated blood platelets to fibrinogen in the presence of LCE used at the whole range of tested concentrations decreased by 9.98 ± 4.51% to 31.50 ± 5.09%. The inhibitory effect of LCE was concentration-dependent and the observed differences were statistically significant (Figure 7). Because of platelet adhesion to Fg/Fb mediates their aggregation and thus activation [29,30,31], we demonstrated anti-aggregative and inactivating properties of LCE relative to blood platelets, pointing at the same time on the disruption of Fg–platelet interactions as the most probable mechanism of such motherwort extract activity. This hypothesis has also been supported by the results of testing the LCE effect on the expression of platelet surface receptors as another relevant determinant of these cells activation. The expression of CD62P (P-selectin) and CD41 (GP IIb/IIIa) on ADP-activated platelets was assessed using flow cytometry (representative dot plots for cytometry analysis are presented on Figure 8). There was no impact of LCE on the level of P-selectin expression on ADP-activated cells, but in response to the growing concentration of extract the expression of GP IIb/IIIa on platelet surface was reduced (max. by 35% at 350 µg/mL of LCE; Figure 9). The percentage of platelets expressing GP IIb/IIIa was also significantly reduced (by 11–29%) in the population of these cells after LCE treatment in comparison to the control cells. Moreover, LCE used at the highest concentration (350 µg/mL) significantly reduced also a percentage of platelets with P-selectin expression (Figure 10). Since CD41 is an integrin complex acting as a receptor for fibrinogen [29,31], these results correlate very well with observed decrease in platelet adhesion to fibrinogen in the presence of LCE. Thus, it can be assumed that molecular mechanism of antiplatelet effect of *L. cardiaca* L. extract is based on the disruption of platelets-fibrinogen interaction by LCE ability to reduce GP IIb/IIIa expression and the number of the cells among whole population expressing this fibrinogen receptor.

Blood platelet activation is a basic process of hemostasis but is also involved in some pathological changes, such as cardiovascular disorders (e.g., atherosclerosis, thrombosis, IE). Intensified blood platelet aggregation is noted in obese people and diabetics, which increases the risk of CVD. In such cases a therapy with antiplatelet drugs (e.g., aspirin) is usually applied. Dietary recommendations to enrich our food with flavonoids, being biologically active components of many plant products (including tested LCE), are other unconventional but quite popular strategies to reduce CVD risk [15,32,33]. Wright et al. [34] showed that such flavonoids as quercetin, apigenin, tamarixetin, and galangin were able to inhibit the aggregation of platelets in plasma (PRP). Polyphenol-rich extracts from berries of *Aronia melanocarpa* (Michx.) Elliott (black chokeberry) and from grape seeds reduced blood platelet adhesion to collagen and fibrinogen, the platelet aggregation and superoxide anion radicals production at in vitro model of hyperhomocysteinemia, which suggests their protective potential [35]. Rahman et al. [36] showed antiplatelet activity of aged garlic extract, which inhibited the binding of activated platelets to fibrinogen and thus preventing their shape changes by increasing in the amounts of cyclic nucleotides (cGMP, cAMP) as intracellular signaling molecules. Epidemiological in vivo studies on the diet effect on CVD demonstrated that consumption of fruit and berries by elderly men impacted on the intima–media thickness (IMT) of the carotid artery and may be protective against carotid atherosclerosis [37]. Since recently, Fruitflow containing water-soluble tomato extract as a source of lycopene able to inhibit platelet aggregation stimulated by ADP or collagen has been commercially available in Europe as the first natural product with cardioprotective action [38]. The results of our in vitro and ex vivo studies indicate relevant *L. cardiaca* L. extract ability to change adhesive properties of staphylococci and to prevent of platelet activation, which may be beneficial in vivo to protect the host against the development of serious infection-associated cardiovascular disorders such as vegetations growing or intravascular coagulation. Moreover, in previously published works on health-promoting effect of plant-origin preparations mainly attention has been paid to their antioxidant properties [35,37,38], while we demonstrated other mechanisms of motherwort extract favorable activity (the modifications of eukaryotic cell surface receptors expression and microbial membranes composition). However, the concentrations of herbal products in practice achieved in vivo are probably the most difficult problem to solve to determine their pro-health doses. After our in vitro and ex vivo studies we can only point to certain range of concentrations, which should be exceeded to exert positive effects, e.g., above 50 µg/mL LCE will be health-promoting because of the antiplatelet effect. We still do not know how to achieve such concentrations in target tissues. Moreover, since the activity of even a single compound depends on many factors (e.g., the structure, time of platelets exposure, presence of plasma proteins) [34], in vivo testing such complex preparations as LCE to assess their bioavailability, pharmacodynamics and pharmacokinetics seem to be necessary.

## 3. Materials and Methods

### 3.1. Bacterial Strain

*Staphylococcus aureus* 8325-4 derived from NCTC 8325, ATCC 35556 parent strain, was used. The strain has been described as expressing of surface-associated adhesive molecules such as MSCRAMMs (microbial surface components recognizing adhesive matrix molecules), SpA (staphylococcal protein A), and ClfA (clumping factor A). Bacteria from frozen stock were grown for 24 h at 37 °C on Tryptic Soy Agar (TSA; BTL, Poland) as a primary culture. Then ready-to-use culture at a required density was freshly prepared in Tryptic Soy Broth (BTL, Poland) with 0.25% glucose (TSB/Glu) using spectrophotometer (Densi-La-Meter II, Erba Lachema, Czech Republic).

### 3.2. Platelets

The study involved seven healthy volunteers and was conducted according to the approval of the University Commission for Bioethics Research No. KBBN-UŁ/II/26/2012. The interview before the study concerned the lack of taking any anticoagulants for 30 days and the exclusion of thrombotic disorders. Blood taken from the ulnar vein was collected into tubes with sodium citrate and centrifuged for 15 min at 190× *g* at 37 °C to obtain platelet rich plasma (PRP).

### 3.3. Preparation of L. cardiaca L. Extract (LCE) Solutions

Commercially available *L. cardiaca* L. (Motherwort) basic plant material (KAWON-HURT Nowak Sp.j., Gostyń, Poland) was extracted and chemically characterized as we previously described [14]. Stock solution of polyphenol-enriched LCE was prepared in 50% DMSO and then diluted in appropriate liquid media up to the concentrations used in each experiment.

### 3.4. LCE Impact on S. Aureus Adhesive Properties Tested Using Atomic Force Microscopy (AFM)

*S. aureus* 8325-4 cells obtained by centrifugation (2500 rpm/10 min) from 1 mL of microbial suspension at OD_535_ = 1.8 were exposed to LCE (0.5 mL) at a concentration of 4.5 mg/mL, which correspond with 0.75 × MIC (minimum inhibitory concentration) and 3 mg/mL (0.5 × MIC) for 24 h at 37 °C. Stock solution of LCE (90 mg/mL in 50% DMSO) was diluted 20 or 30 times in TSB/Glu to obtain the final LCE concentrations (4.5 or 3 mg/mL, respectively). The highest concentration of DMSO as LCE primary solvent reached then 2.5%. Therefore, the control bacteria were prepared in TSB/Glu containing 2.5% DMSO to exclude any solvent effect on microbial viability. After exposure, bacteria were centrifuged (in conditions as above), suspended in 0.5 mL fresh TSB/Glu and supplied to the Center of Quantum Optics at the Faculty of Physics, Astronomy and Informatics, Nicolaus Copernicus University in Toruń (Poland) to perform AFM measurements.

*S. aureus* treated with LCE and untreated (control) were immobilized on poly-l-lysine (PLL, Sigma-Aldrich, Saint Louis, MO, USA) coated glass surfaces. Cell suspensions in phosphate-buffered saline (PBS) were deposited for 45 min. on PLL-coated slides. Loosely bound cells were removed by thorough rinsing with fresh Milli-Q water. Samples were used immediately for force spectroscopy experiments. AFM measurements were performed in PBS with a Bioscope II AFM equipped with a NanoScope V controller (Veeco) and using an MLCT-D silicon nitride cantilever with a nominal tip apex radius of 20 nm. The cantilevers were calibrated prior to the experiments by a thermal noise method, an average spring constant was 0.03 N/m. To localize individual cells, images were obtained in an intermittent contact mode. Force spectroscopy measurements were then performed in the contact mode. At least 5 force-distance curves (each curve in a different location) were collected for each within a batch of 30–50 different bacterial cells, using a maximum applied force of 1 nN, a constant retraction speed of 2 µm/s and retract delay 1 s. Adhesion forces were calculated as described before [39]. Control force–distance curves were registered on bacteria-free area before and after proper measurements on bacterial cells to confirm that the AFM tip had not been contaminated by bacterial biopolymers. Force curves were analyzed in NanoScope Analysis 1.7 software (Bruker, Billerica, MA, USA). The effect of the LCE on bacteria cell were obtained in contact mode in air. The topography and deflection images were obtained simultaneously at a can rate of 0.5 Hz at a resolution of 512 pixel per line on 5 × 5 µm^2^ area. The data were analyzed with Gwyddion 2.47 software [40].

### 3.5. The Effect of LCE on Staphylococcal Cell Membrane Lipid Profile

*S. aureus* 8325-4 cells obtained by centrifugation (2500 rpm/10 min) from 8 mL of microbial suspension at OD_535_ = 1.8 were exposed to LCE (8 mL) at a concentration of 4.5 mg/mL (0.75 × MIC) and 3 mg/mL (0.5 × MIC) for 24 h at 37 °C with shaking. Stock solution of LCE (90 mg/mL in 50% DMSO) was diluted as described in Section 3.4. The highest concentration of DMSO as primary solvent reached then 2.5%. Therefore, the control bacteria were prepared in TSB/Glu containing 2.5% DMSO to exclude any solvent effect on microbial viability. Then, each sample was divided into 4 tubes of 2 mL and analyzed by chromatography and mass spectrometry as described below.

#### 3.5.1. Chemicals

The following reagents were used: 1,2-dimyristoyl-sn-glycero-3-phospho-rac-(1-glycerol) (sodium salt) (14:0/14:0 PG); 1,2-dilauroyl-sn-glycero-3-phosphoethanolamine (12:0/12:0 PE) and 1’,3’-bis[1,2-dimyristoyl-sn-glycero-3-phospho]-sn-glycerol (sodium salt) (14:0 Cl) purchased from Avanti Polar Lipids (Alabaster, AL, USA). The other chemicals were acquired from Sigma-Aldrich (Saint Louis, MO, USA), and POCh (Gliwice, Poland). All chemicals were high purity grade reagents.

#### 3.5.2. Phosholipid Extraction and HPLC Analysis

Phospholipids (PLs) from *S. aureus* suspensions were extracted according to the method proposed by Folch et al. [41], with some modifications. The bacterial biomass was transferred into Eppendorf tubes containing glass beads, 0.66 mL of chloroform and 0.33 mL of methanol. The homogenization process using a ball mill (FastPrep) was carried out for 1 min. The mixture was extracted for 2 min. In order to facilitate the separation of two layers, 0.2 mL of 0.9% saline was added. The lower layer was collected and evaporated. The phospholipid extracts were dissolved in 750 μL of chloroform: methanol (1:9, *v/v*) solution.

The polar lipids were measured using an Agilent 1200 HPLC system (Santa Clara, CA, USA) and a 4500 QTRAP mass spectrometer (Sciex, Torrance, CA, USA) with an ESI source. For the reversed-phase chromatographic analysis, 10 μL of the lipid extract was injected onto a Kinetex C18 column (50 mm × 2.1 mm, particle size: 5 μm; Phenomenex, Torrance, CA, USA). The mobile phase consisted of 5-mM ammonium formate in water (A) and 5-mM ammonium formate in methanol (B). The solvent gradient was initiated at 70% B, increased to 95% B over 1.25 min, and maintained at 95% B for 16 min before returning to the initial solvent composition over 3 min. The column temperature was maintained at 40 °C, and the flow rate was 500 µL/min. The instrumental settings were as follows: spray voltage −4.500 V, curtain gas (CUR) 25, nebulizer gas (GS1) 50, turbo gas (GS2) 60, and ion source temperature of 600 °C. The data analysis was performed with the Analyst™ v1.6.2 software (Sciex).

Two approaches were applied to identify PLs: targeted and untargeted. The untargeted approach was performed with the precursor ion scanning (precursor for *m/z* 153) survey scan, triggering the EPI experiments. On the basis of the untargeted analysis, a comprehensive list of the multiple reaction monitoring (MRM) transitions was generated (parent fatty acyl fragment) for the following PL classes: phosphatidylglycerol (PG), lysyl-phosphatidylglycerol (LPG), and cardiolipin (CL).

The LC-MS/MS method was validated with the standards of (14:0/14:0 PG), (12:0/12:0 PE) and (14:0 CL). The LOD range of phospholipid reference compound varied between 3 ng/mL for PG, 5 ng/mL for PE, and 35 ng/mL for CL. CL was the most difficult lipid to quantify, since the shape of the peaks. The lipids belonging to each class in examined samples were quantified upon comparison with the standard of the relevant class. The percent coefficient of variation was less than 22% for all phospholipids.

#### 3.5.3. Fatty Acids Extraction and GC-MS Analysis

*S. aureus* cell pellet in Eppendorf tube was diluted in mixture of methanol (0.75 mL), toluene (0.1 mL), and 8.0% HCl solution in methanol (0.15 mL) [42]. The tube was vortexed and then incubated overnight at 45 °C. After cooling to room temperature, 1 mL of hexane and 1 mL of water were added for the extraction of fatty acid methyl esters (FAMEs). The tube was vortexed, and then 0.3 mL of hexane layer was moved to the chromatographic vial. 1 µL of the extract samples were analyzed using gas chromatography.

FAMEs analysis was performed with a Agilent Model 7890 gas chromatograph, equipped with a 5975C mass detector. The separation was carried out in the capillary column HP 5 MS methyl polysiloxane (30 m × 0.25 mm i.d. × 0.25 mm ft). The column temperature was maintained at 60 °C for 3 min, then increased to 212 °C at the rate of 6°C/min, followed by an increase to 245 °C at the rate of 2 °C/min, and finally to 280 °C at the rate of 20 °C/min. The column temperature was maintained at 280 °C for 10 min. Helium was used as the carrier gas at the flow rate of 1 mL/min. The injection port temperature was 250 °C. Split injection was employed. Bacterial fatty acids were identified by comparison with the retention times of the authentic standards (Sigma, Supelco, Sigma-Aldrich) or by their mass spectra, and finally, the results were expressed as a percentage of the total amount of fatty acids.

### 3.6. LCE Influence on Blood Platelet Adhesion to Fibrinogen

The suspension of blood platelets (1 × 10^8^ cells/mL) treated with LCE at concentration of 50, 100, and 350 µg/mL or untreated (control) were applied to the wells of culture 96-well plate (Nunc, Denmark) previously coated with fibrinogen as described by Micota et al. [14]. The samples were incubated for 1 hour at 37 °C, 5% CO_2_. Then the wells were washed with 200 μL of TBS followed by addition of 50 mL lysis buffer for 10 min (stirring constantly). The supernatants (25 μL) were transferred to a new 96-well plate to assess the protein concentration using colorimetric Bicinchoninic Acid Protein Assay Kit (Sigma).

### 3.7. Flow Cytometric Analysis of Blood Platelets Activation after Exposure to LCE

PRP was exposed on LCE at a concentration of 50, 100, and 350 µg/mL with the highest DMSO concentration of 0.2%, which has no effect of cell viability. To the control PRP Tris-buffered saline (TBS; 50 mM Tris-HCl, 150 mM NaCl, pH 7.6) was added. All samples were stimulated with 20 mM ADP (Sigma) at 37 °C for 5 min. Then the samples were transferred to cytometric tubes and 10 μL mouse monoclonal Ab anti human CD62P (P-selectin) labeled with AlexaFluor 488 (Bio-Rad, Hercules, CA, USA) and 10 μL mouse monoclonal Ab anti human CD41 (GP IIb/IIIa) labeled with PE (Bio-Rad) were added. The required isotypic controls were included. The samples were incubated for 30 min on ice and then analyzed using LSR II Digital Analytical Flow Cytometer (Becton Dickinson, Franklin Lakes, NJ, USA).

### 3.8. Statistics

The results were analyzed for significance using nonparametric Kruskal–Wallis one-way ANOVA test and the program Statistica 12.0 (Stat Soft Inc., Tulsa Shock, OK, USA). The differences with *p* < 0.05 were considered to be statistically significant.

## 4. Conclusions

The results of our in vitro and ex vivo studies demonstrated essential *L. cardiaca* L. extract biological activity against both staphylococcal adhesion and blood platelet activation, confirming its pro-health potential. Molecular mechanism of LCE antiplatelet effect was based on the disruption of platelet–fibrinogen interactions by altering GP IIb/IIIa expression. We also showed the effect of LCE on the morphology of *S. aureus* cells, as well as the composition of phospholipids and fatty acids in their membranes, which may both positive and negative consequences. Thus, by an application of LCE (e.g., as an addition to the daily diet) staphylococcal adhesion, aggregation, and biofilm formation could be inhibited. However, the resistance of these bacteria to some antibiotics may be simultaneously enhanced, which indicates the necessity of exclusion the cationic antibiotics (e.g., daptomycin) from infection treatment, when motherwort herb is used at the same time. Therefore, improved understanding the mechanisms of LCE activity may serve to develop proper recommendations for the use of herbal products based on *L. cardiaca* L. extracts as preventive preparations or supportive products for classic antibiotics in cases of infective endocarditis and other invasive infections.
